# High-Performance Polymer Dispersed Liquid Crystal Enabled by Uniquely Designed Acrylate Monomer

**DOI:** 10.3390/polym12081625

**Published:** 2020-07-22

**Authors:** Rijeesh Kizhakidathazhath, Hiroya Nishikawa, Yasushi Okumura, Hiroki Higuchi, Hirotsugu Kikuchi

**Affiliations:** Institute for Materials Chemistry and Engineering, Kyushu University, Fukuoka 819-0395, Japan; hiroya.nishikawa@riken.jp (H.N.); okumura@cm.kyushu-u.ac.jp (Y.O.); higuchi@cm.kyushu-u.ac.jp (H.H.)

**Keywords:** liquid crystals, monomers, polymerization, electro–optical properties

## Abstract

The widespread electro–optical applications of polymer dispersed liquid crystals (PDLCs) are hampered by their high-driving voltage. Attempts to fabricate PDLC devices with low driving voltage sacrifice other desirable features of PDLCs. There is thus a clear need to develop a method to reduce the driving voltage without diminishing other revolutionary features of PDLCs. Herein, we report a low-voltage driven PDLC system achieved through an elegantly simple and uniquely designed acrylate monomer (A3DA) featuring a benzene moiety with a dodecyl terminal chain. The PDLC films were fabricated by the photopolymerization of mono- and di-functional acrylate monomers (19.2 wt%) mixed in a nematic liquid crystal E7 (80 wt%). The PDLC film with A3DA exhibited an abrupt decline of driving voltage by 75% (0.55 V/μm) with a high contrast ratio (16.82) while maintaining other electro–optical properties almost the same as the reference cell. The response time was adjusted to satisfactory by tuning the monomer concentration while maintaining the voltage significantly low (3 ms for a voltage of 0.98 V/μm). Confocal laser scanning microscopy confirmed the polyhedral foam texture morphology with an average mesh size of approximately 2.6 μm, which is less in comparison with the mesh size of reference PDLC (3.4 μm), yet the A3DA-PDLC showed low switching voltage. Thus, the promoted electro–optical properties are believed to be originated from the unique polymer networks formed by A3DA and its weak anchoring behavior on LCs. The present system with such a huge reduction in driving voltage and enhanced electro–optical performance opens up an excellent way for abundant perspective applications of PDLCs.

## 1. Introduction

Polymer dispersed liquid crystals (PDLCs) have attracted considerable attention for a large range of electro–optical applications, including flat panel displays, smart windows, light shutters, and holographic films [1,2,3,4,5,6,7,8,9,10,11,12,13]. Employing PDLCs in technologies is a highly promising approach due to their easy processability, large area coverage, and low cost. The micrometer-sized liquid crystal droplets dispersed in a polymer matrix are referred to as PDLC, which can be switched from a translucent (OFF) state to a transparent (ON) state by the application of an electric field [14,15,16,17,18,19]. The electric field induces the re-orientation of randomly distributed liquid crystal directors in domains in the opaque state to a preferential direction parallel to the applied field and opto-electric switching occurs if the refractive index of liquid crystal matches with that of polymer matrix [20,21]. The electro–optical properties of PDLCs therefore mainly depend on liquid crystalline material, chemical nature of polymer as well as the polymerization conditions [22,23].

The principal strategy to prepare PDLC is a phase separation process between mixtures of LCs and polymers [24,25,26]. The major methodologies are thermally induced phase separation (TIPS), polymerization-induced phase separation (PIPS) and solvent-induced phase separation (SIPS) [27,28,29,30,31,32]. Among these, photopolymerization based PIPS is far more favorable in terms of convenience, simplicity, and the offered control over the morphology of PDLC [33].

The PDLCs have several advantages, which include no requirement of polarizers, flexibility, high transmittance, short open time, [14,16,34,35] however, they typically suffer from high voltage, poor contrast ratio, off-axis haze, etc. Tremendous efforts were progressed previously on optimization of PDLC materials and structure of devices to fabricate low voltage PDLCs with enhanced electro–optical properties and were sufficiently impressive [20,21,36,37,38,39,40,41]. Despite promising results, attempts at constructing low-voltage driven PDLCs have involved either cumbersome fabrication procedures or compromised on other salient features of PDLCs [36,37,38,39,40,41,42,43,44,45,46,47,48]. There is therefore high interest in developing an efficient method to reduce the driving voltage of PDLCs without sacrificing other desirable features.

Herein, we report an elegantly simple and efficient strategy to achieve remarkable driving voltage reduction in PDLCs based on a simple and uniquely designed mono-functional acrylate monomer, A3DA. This monomer features a dodecyl group at the para position of phenyl ring attached to a polymerizable acrylate part with a propyl spacer. The PDLC system built on A3DA showed an abrupt decline of driving voltage for about 75% while preserving the other electro–optical properties of PDLCs more or less the same. Such a huge reduction in driving voltage, coupled with the revolutionary features of PDLCs, makes them more viable as smart materials from an application point of view.

## 2. Experimental Section

### 2.1. Materials

All chemicals were obtained from commercial sources and used without further purification, unless otherwise stated. Liquid crystal cells with a cell gap of 10 μm were purchased from E.H.C. Co., Ltd (Tokyo, Japan).

### 2.2. Synthesis of A0DA and A3DA

See reference [49].

### 2.3. Sample Preparation

**Figure 1:**
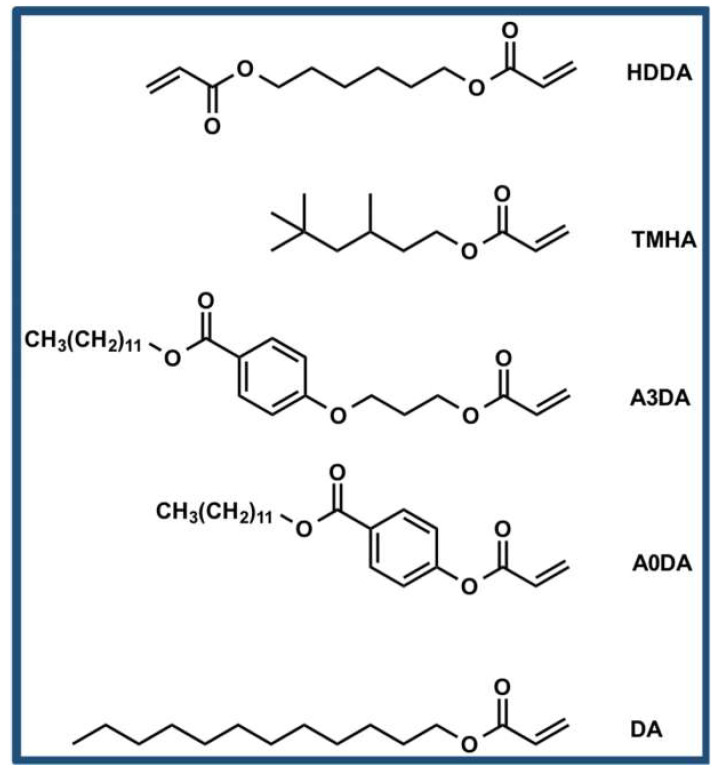
Chemical structure of monomers used to prepare polymer dispersed liquid crystals (PDLCs).

Molecular structures of monomers used to prepare PDLC films are shown in Figure 1. The PDLC precursor mixture contains liquid crystals E7 (80 wt%), a mono-functional monomer benzoic acid, 4-[3-[(1-oxo-2-propen-1-yl)oxy]propoxy]-,dodecyl ester (A3DA) or 3,5,5-trimethylhexylacrylate (TMHA) or benzoic acid, 4-[(1-oxo-2-propen-1-yl)oxy]-,dodecyl ester (A0DA) or dodecyl acrylate (DA) (9.6 wt%), a cross-linker 1,6-hexanediol diacrylate (HDDA) (9.6 wt%), and a photoinitiator 2,2-imethoxy-2-phenylacetophenone (DMAP) (0.8 wt%) were stirred at 30 °C for 30 min, then injected into a 10-μm-thick ITO coated liquid crystal cells (E. H. C. Co., Ltd.) by capillary action. Subsequently, the mixture was irradiated with UV light at 365 nm with an intensity of 1.5–2 mW cm^−2^ for 30 min at 30 °C and the electro–optical properties were measured. Liquid crystal cells made of glass plate and cover slip with a cell thickness of 10-μm were used for confocal laser scanning microscopy. Samples with dye concentration of 0.05% and 0.1% by weight were prepared and polymerized under the identical conditions to corroborate the consistency.

### 2.4. Measurements

The samples were observed using a Nikon Eclipse LV 100 POL optical microscope equipped with a Nikon DS-Ri1 CCD camera and a Linkam hot stage (10013L). The electro–optical properties were measured on an experimental set up shown in Figure S1 (see Supplementary Materials). For the transmittance calculation, the transmittance of air was normalized as 100%. The transmittance of sample cells was determined by applying a square-wave AC voltage of 1 kHz. All the electro–optical measurements were carried out at 303 K. The voltages corresponding to 10% and 90% of transmittance between the minimum and maximum transmittances were defined as threshold voltage (Vth) and the saturation voltage (Vmax), respectively. The morphology of the polymer network in PDLC films was determined using a fluorescent confocal microscope (Nikon A1).

## 3. Results and Discussion

**Figure 2:**
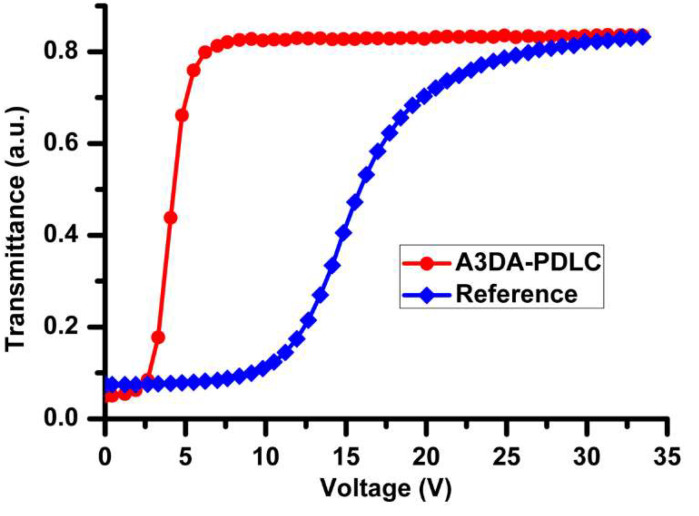
Transmittance dependence on the applied electric field for A3DA-PDLC (sample B) and reference cell (sample R) measured at 303 K, λ = 633 nm.

**Figure 3:**
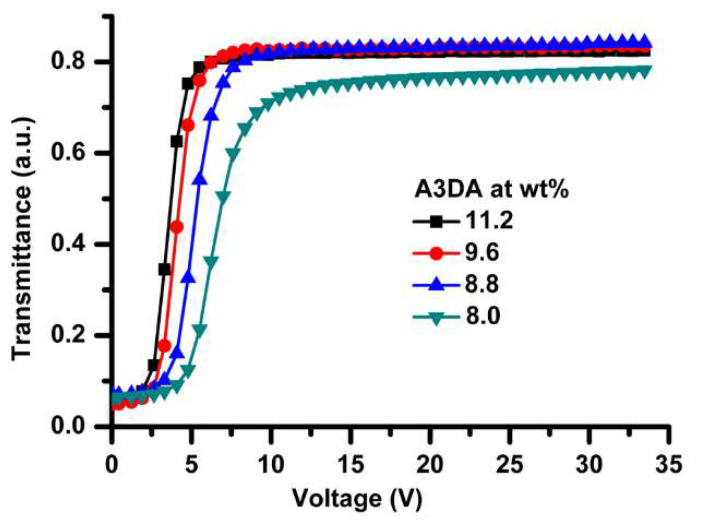
Voltage-transmittance (V-T) curves for PDLCs prepared with A3DA at different concentrations at 303 K, λ = 633 nm.

The PDLC films were made according to the procedure outlined in the experimental section. A variety of mixing ratios of monomers (Table 1) while modulating the total concentration of monomers to 19.2 wt% was employed to optimize the overall PDLCs performances. A reference device was also fabricated for comparison. The weight fractions of liquid crystal (E7) and photoinitiator (DMPAP) were invariable for all the experiments. The voltage-dependent-transmittance (V-T) characteristics of PDLC films were measured by applying a square wave AC voltage of 1 kHz frequency and the typical V-T characteristics obtained for these samples are shown in Figure 2 and Figure 3. The PDLC films were milky when no electric-field was applied. The transmittance increased with increasing applied voltage and reached maximum value at the saturation field and reverted to the scattering state when the voltage is removed. Interestingly, the PDLC film that contains A3DA showed a huge reduction in driving voltages, regardless of the percentage composition of the monomer present, compared to the reference cell (Figure 2).

The reference cell exhibited a Vmax of 22.8 V (Vth = 11.2 V). Pleasingly, the V-T curves of PDLC containing A3DA presented an unexpected voltage reduction for about 75%, from 22.8 to 5.5 V (Vth = 2.7) at an analogous blending ratio to that of reference cell (Figure S2, Supplementary Materials). Decreasing A3DA content resulted in an increase of Vmax slightly to 7.6 and 9.8 V for 8.8 wt% and 8.0 wt% A3DA blends, respectively (Figure S3 and Table S1, Supplementary Materials). Whilst the impact of increasing A3DA concentration on voltage reduction was negligible, the decreased amount of cross-linker resulted in memory effect—a situation supported by the appearance of increased transparency even after the film was fully switched to OFF state (Figure S3, Supplementary Materials) [50].

**Figure 4:**
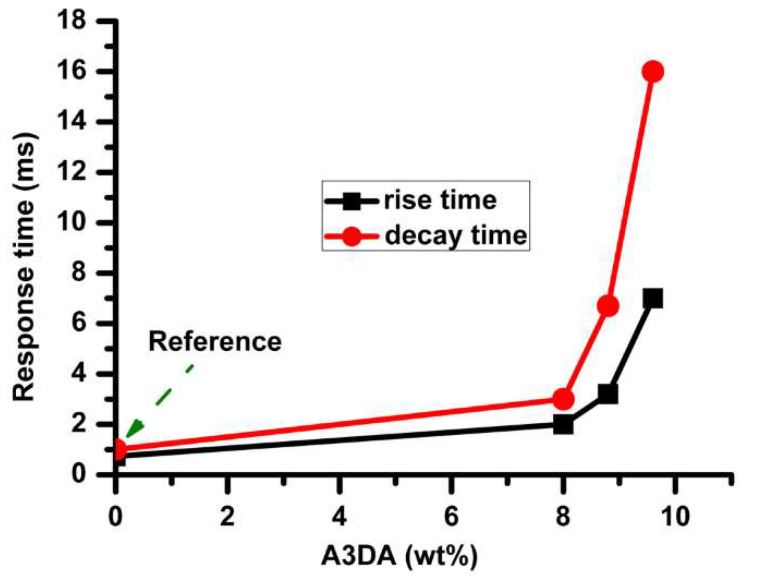
Response times for PDLC films with varied content of A3DA and reference cell.

We then studied the effect of A3DA on response times. Figure 4 shows the comparison of response time between the samples containing A3DA and reference device with an applied square wave electric filed (f = 1 kHz). The decay time of the reference cell was 1 ms, whereas a slower response was observed for PDLC prepared with 9.6 wt% A3DA (sample B) with a decay time of 14 ms. When the A3DA concentration was 8.8 wt% (sample C), the decay time drops off to 6.7 ms with a slight increase in driving voltage (7.6 V). Such decreasing in the decay time was improved to 3 ms with a further reduction in the A3DA mixing ratio to 8 wt% (sample D). The rise times were a few to 7 ms and followed a decreasing tendency with decreasing amounts of A3DA (see Supplementary Materials, Table S1). Further optimization studies, at concentrations lower than 8.0 wt% were not carried out due to the increase of Vmax with low A3DA fraction. These ratios could be further optimized to have ultra-fast low voltage driven PDLC devices and such studies are currently underway.

Contrast ratio (CR) is a measure of transmittance between the transparent state and opaque state and is of great importance to the performance of the PDLC system [20]. The PDLC film containing 9.6 wt% A3DA by weight was characterized by a high contrast ratio (16.82), which was better than that shown by reference cell (11.30). The contrast ratio of other samples was more or less similar compared to sample R (Table S1, Supplementary Materials). The scattering of light in the OFF state depends on the LC droplet size, whereas ON state transmittance reflects the good match of the refractive index between the LC and polymer matrix. Thus, a high contrast ratio could be a consequence of the formation of appropriate polymer networks enabled by our unique monomer A3DA.

**Figure 5:**
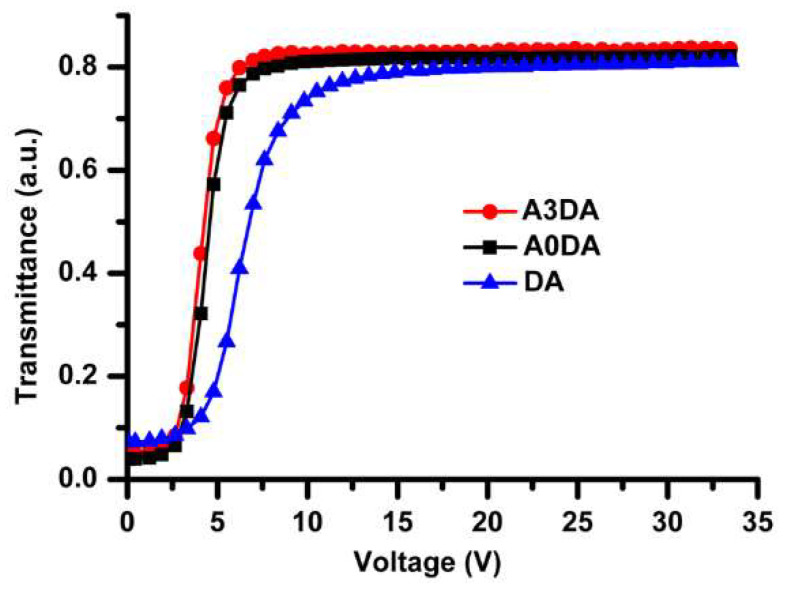
V-T curves for PDLC films made of different monomers.

The driving voltage reduction of polymer network liquid crystal containing 1–2% of DA has been reported to originate from the weakening of interface anchoring strength [51]. The PDLC film containing DA was therefore fabricated to investigate the effect of the terminal chain on the observed excellent electro–optical (EO) performances of A3DA-PDLCs. Interestingly, a huge voltage reduction with a Vmax value of 10.5 V was observed for DA-PDLC also (Figure 5, see Figure S4 and Table S2 in Supplementary Materials for more information). This result confirms that the dodecyl end group contributes significantly to the lowering of driving voltage in A3DA-PDLC. However, the presence of the dodecyl group in A3DA cannot solely justify the observed remarkable voltage reduction in PDLCs constructed with A3DA.

We then fabricated PDLC devices with monomer A0DA (Figure 5), while keeping the mixing ratio constant (9.6 wt%) to elucidate the impact of a spacer on V-T characteristics of PDLCs. A0DA is structurally similar to A3DA but does not possess a spacer unit. The dependence of transmittance on voltage was similar (Vmax = 6.2 V) for PDLC containing A0DA to that of obtained for A3DA-PDLC (Figure 5), which negates the effect of a spacer on lowering of driving voltage in A3DA-PDLC. Based on the above results, it is clear that some other factors also need to be considered for the adequate understanding of the promoted electro–optical properties of A3DA based PDLCs.

There are many factors that could affect the electro–optical performances of PDLC systems that include size and shape of the LC domains, nature of the polymer matrix, anchoring energy of liquid crystal domains, characteristics of LC, and thickness of the film [14]. In this study, we prepared PDLC cells of the same thickness under similar experimental conditions and E7 was chosen as the LC component for all the experiments. Thus, the elements determining the EO efficiencies of PDLC with A3DA were limited to the morphology of LC droplets and interaction between the LC molecules and the polymer network.

To examine the morphology of PDLC films, confocal laser scanning microscopy (CLSM) was carried out. For this inspection, PDLC precursor mixtures were added with a small amount of acridine yellow (0.05–0.1 wt%) based mono-functional monomer dye to selectively concentrate in polymer networks of resulting PDLC samples. All other conditions of preparation were kept identical as detailed before. The structure of the monomer and the film morphology are a subject of another study in our group and hence are not disclosed in detail in this paper.

**Figure 6:**
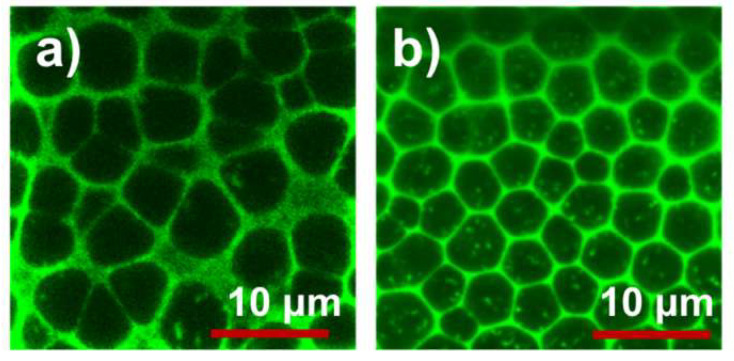
Confocal microscopy images of reference cell (**a**) and A3DA-PDLC film (**b**) with 0.1 wt% of dye. The images were collected near the film-cover glass interface (UV light exposed area).

CLSM images of A3DA and reference PDLC cells revealed a polyhedral foam texture morphology (Figure 6, also see Figure S5 in Supplementary Materials) [52]. Whilst reference cell morphology was non-uniform, the PDLC film made of A3DA resulted in a monodispersed droplets morphology with an average mesh size of approximately 2.6 μm, which is less than that observed for reference cell, 3.4 μm. The smaller droplet size in A3DA-PDLC may have originated from the enhanced chemical affinity between A3DA bearing benzene ring and LC molecules owing to their chemical structure similarity. An alternative reason for smaller LC domains could be the increase in viscosity of PDLC composites attributed to the increase in chain rigidity of the A3DA monomer. When the viscosity of the system increases, the molecular diffusion rate of monomer free radicals and coalescence rate of LC domains decreases and results in the formation of smaller droplets. This result reveals a relatively higher OFF state optical scattering of A3DA-PDLC.

The polydispersity of droplet size and shape usually broadens the V-T curves as the droplets of different size clear at different field strengths and the transmittance reaches a maximum value gradually [52,53]. In contrast, the PDLCs with drop size uniformity are expected to follow a square jump in its electro–optical performance and show steep V-T curves [52,53]. The observed field-dependent transmittance characteristics of A3DA and reference PDLC cells agree well with the aforesaid points.

Generally, a decrease in mesh size causes an increase in the surface anchoring energy of LC, which in turn increases the driving voltage of the system [52,54]. However, the present system showed an abrupt decline of operating voltage with a reduced average mesh size of LC droplets. This can be possible only if the anchoring energy between the polymer and LC is weaker. When the anchoring energy between the LC and polymer is weak, LC molecules respond to an applied electric field easily and thereby reduce the voltage required to switch the system [55,56]. Under such a condition, however, the LC molecules recover to its original state slowly after the removal of the applied field, resulting in the increase of decay time. These interpretations are consistent with our experimental results. The PDLCs prepared with A3DA and DA exhibited voltage reduction with an increase of decay time in comparison with the reference cell. However, the driving voltage reduction and decay time increase from DA- to A3DA-PDLCs, suggesting that LC molecules experience much weaker anchoring in the polymer network formed by A3DA and HDDA than the polymer system formed by DA and HDDA. Also, decreasing of A3DA concentration results in improvements of decay time. This provides further evidence of the weak anchoring nature of A3DA. We have recently reported that the switching voltages could be reduced for blue phase liquid crystals stabilized with A3DA because of the decrease in the interface anchoring strength [49]. Taking into consideration all of the data mentioned above, it is reasonable to consider that the driving voltage reduction in PDLC prepared with A3DA and HDDA probably resulted from the reduction of anchoring energy between polymer networks and LCs enabled by the uniquely designed A3DA monomer. However, it is unclear whether the observed remarkable voltage reduction in A3DA-PDLC is specific to weak anchoring energy, or if some other factors are involved.

The present system has advantages over the conventional PDLC system based on alkyl acrylate mixtures. The benzene ring in A3DA enhances the solubility of starting mixture due to the increased compatibility with LC molecules, whereas the dodecyl moiety maintains the miscibility gap between LCs and growing polymer matrix to generate a two-phase structure that can strongly scatter the light. The feature of the monomer controls the morphology of the system and at the same time decreases the interface anchoring energy of the interwoven polymer network on LCs, which thus results in enhanced electro–optical performance of the PDLC films. We expect that such a unique and simple design will be a promising candidate for various applications based on polymer–liquid crystal composites.

## 4. Conclusions

In conclusion, we present a highly efficient low-voltage driven PDLC system based on a simple and uniquely designed acrylate monomer bearing a benzene ring, propyl spacer, and dodecyl tail. The optimized material showed a remarkable decline of driving voltage with a high contrast ratio, while preserving other electro–optical properties. The response time could be adjusted to a desirable limit by tuning A3DA concentration. We believe that such a huge decrease in operating voltage for PDLCs constructed with A3DA possibly resulted from the weakening of anchoring energy between LC molecules and polymer networks containing A3DA. We anticipate that the development of a highly efficient and low-voltage driven polymer/LC dispersed system, as presented here, could have a broad impact on applications, such as smart windows, displays, holographic PDLCs, etc.

## Figures and Tables

**Table 1:** Chemical compositions of the PDLC precursors.

Sample Name	E7 (80 wt%), DMPAP (0.8 wt%)		
	A3DA (wt%)	HDDA (wt%)	TMHA
R (Std.)	-	9.6	9.6
A	11.2	8	-
B	9.6	9.6	-
C	8.8	10.4	-
D	8.0	11.2	-

## Supplementary Materials

The following are available online at https://www.mdpi.com/2073-4360/12/8/1625/s1. Figure S1: Experimental set up for electro-optical properties measurements of PDLCs; Figure S2: V-T curves for forward (blue circles) and backward (red circles) processes of A3DA-PDLC and reference cell; Figure S3: V-T curves for forward (blue circles) and backward (red circles) processes of A3DA-PDLC at various concentrations. a) 8.0 wt%, b) 8.8 wt%, c) 9.6 wt%, d) 11.2 wt%; Figure S4: V-T curves for forward (blue circles) and backward (red circles) processes of A0DA-PDLC (a) and DA-PDLC (b); Figure S5: Confocal microscopy images of reference cell (a) and A3DA-PDLC (b) with 0.05 wt% of dye. The images were collected near the film-cover glass interface (UV light exposed area); Table S1: EO properties of PDLCs prepared with A3DA and reference cell at 303 K, λ = 633 nm; Table S2: EO properties of PDLCs prepared with A3DA, A0DA and DA at 303 K, λ = 633 nm. The monomer mixing ratio was 9.6 wt%.

## References

[1] Oh S.-W., Baek J.-M., Heo J., Yoon T.H. (2016). Dye-doped cholesteric liquid crystal light shutter with a polymer-dispersed liquid crystal film. Dye. Pigment..

[2] Geis M., Lyszczarz T.M., Osgood R.M., Kimball B.R. (2010). 30 to 50 ns liquid-crystal optical switches. Opt. Express.

[3] Wu B.-G., Erdmann J.H., Doane J.W. (1989). Response times and voltages for PDLC light shutters. Liq. Cryst..

[4] Wang K., Zheng J., Liu Y., Gao H., Zhuang S. (2017). Electrically tunable two-dimensional holographic polymer-dispersed liquid crystal grating with variable period. Opt. Commun..

[5] Chen G., Ni M., Peng H., Huang F., Liao Y., Wang M.-K., Zhu J., Roy V.A.L., Xie X. (2017). Photoinitiation and Inhibition under Monochromatic Green Light for Storage of Colored 3D Images in Holographic Polymer-Dispersed Liquid Crystals. ACS Appl. Mater. Interf..

[6] Sheraw C., Zhou L., Huang J.R., Gundlach D.J., Jackson T.N., Kane M.G., Hill I.G., Hammond M.S., Campi J., Greening B.K. (2002). Organic thin-film transistor-driven polymer-dispersed liquid crystal displays on flexible polymeric substrates. Appl. Phys. Lett..

[7] Cong S., Cao Y., Fang X., Wang Y., Liu Q., Gui H., Shen C., Cao X., Kim E.S., Zhou C. (2016). Carbon Nanotube Macroelectronics for Active Matrix Polymer-Dispersed Liquid Crystal Displays. ACS Nano.

[8] Mach P., Rodriguez S.J., Nortrup R., Wiltzius P., Rogers J.A. (2001). Monolithically integrated, flexible display of polymer-dispersed liquid crystal driven by rubber-stamped organic thin-film transistors. Appl. Phys. Lett..

[9] Kashima M., Cao H., Liu H.J., Meng Q.Y., Wang D., Li F.S., Yang H. (2010). Effects of the chain length of cross-linking agents on the electro-optical properties of polymer dispersed liquid crystal films. Liq. Cryst..

[10] Sahraoui A.H., Delenclos S., Longuemart S., Dadarlat D. (2011). Heat transport in polymer-dispersed liquid crystals underelectric field (Report). J. Appl. Phys..

[11] Hikmet R.A.M., Kemperman H. (1998). Electrically switchable mirrors and optical components made from liquid-crystal gels. Nature.

[12] Yang H., Mishima K., Matsuyama K., Hayashi K.-I., Kikuchi H., Kajiyama T. (2003). Thermally bandwidth-controllable reflective polarizers from (polymer network/liquid crystal/chiral dopant) composites. Appl. Phys. Lett..

[13] Natarajan L.V., Shepherd C.K., Brandelik D.M., Sutherland R.L., Chandra S., Tondiglia V.P., Tomlin D., Bunning T.J. (2003). Switchable Holographic Polymer-Dispersed Liquid Crystal Reflection Gratings Based on Thiol−Ene Photopolymerization. Chem. Mater..

[14] Mucha M. (2003). Polymer as an important component of blends and composites with liquid crystals. Prog. Polym. Sci..

[15] Doane J.W., Bahadur B. (1992). Liquid Crystals. Applications and Uses.

[16] Doane J.W., Vaz N.A., Wu B.-G., Žumer S. (1986). Field controlled light scattering from nematic microdroplets. Appl. Phys. Lett..

[17] Crawford G.P., Zumer S. (1996). Liquid Crystals in Complex Geometries. Adv. Mater..

[18] Fergasen L. (1984). Encapsulated Liquid Crystal and Method. U.S. Patent.

[19] Doane J.W., Chidichimo G., Vaz N.A. (1987). Light Modulating Material Comprising a Liquid Crystal Dispersion in a Plastic Matrix. U.S. Patent.

[20] Sun Y., Zhang C., Zhou L., Fang H., Huang J., Ma H., Zhang Y., Yang J., Zhang L.-Y., Song P. (2016). Effect of a Polymercaptan Material on the Electro-Optical Properties of Polymer-Dispersed Liquid Crystal Films. Molecules.

[21] Ellahi M., Liu F., Song P., Gao Y., Rafique M.Y., Khan D.F., Cao H., Yang H. (2014). Characterization and Morphology of Polymer-Dispersed Liquid Crystal Films. Soft Mater..

[22] Kovalchuk A.V., Kurik M.V., Lavrentovich O.D., Sergan V.V. (1988). Structural transformations in nematic drops located in an external electric-field. Sov. Phys. JETP.

[23] Kovalchuk A.V., Lavrentovich O.D., Sergan V.V. (1989). Kenetic of electrooptical effects in drops of nematic with different structure. Sov. Tech. Phys. Lett..

[24] Srivastava J.K., Singh R.K., Dhar R., Singh S. (2011). Thermal and morphological studies of liquid crystalline materials dispersed in a polymer matrix. Liq. Cryst..

[25] Perju E., Marin L., Grigoras V.C., Bruma M. (2011). Thermotropic and optical behaviour of new PDLC systems based on a polysulfone matrix and a cyanoazomethine liquid crystal. Liq. Cryst..

[26] Cho Y., Kawakami Y. (2006). High performance holographic polymer dispersed liquid crystal systems using multi-functional acrylates and siloxane-containing epoxides as matrix components. Appl. Phys. A.

[27] Kim S.H., Heo C.P., Park K.S., Kim B.K. (1998). Effect of prepolymer structure on the electro-optic performance of polymer dispersed liquid crystals. Polym. Int..

[28] Li W., Cao H., Kashima M., Liu F., Cheng Z., Yang Z., Zhu S., Yang H. (2008). Control of the microstructure of polymer network and effects of the microstructures on light scattering properties of UV-cured polymer-dispersed liquid crystal films. J. Polym. Sci. Part B Polym. Phys..

[29] Tao H., Zhang J., Wang X., Gao J. (2006). Phase separation and polymer crystallization in a poly(4-methyl-1-pentene)–dioctylsebacate–dimethylphthalate system via thermally induced phase separation. J. Polym. Sci. Part B Polym. Phys..

[30] Miyamoto A., Kikuchi H., Kobayashi S., Morimura Y., Kajiyama T. (1991). Dielectric property-electrooptical effect relationships of polymer/liquid-crystal composite films. Macromolecules.

[31] Kikuchi H., Usui F., Kajiyama T. (1996). Control of Phase-Separated Structure in (Polymer/Liquid Crystal) Composite Films and Their Electro-Optical Switching Characteristics. Polym. J..

[32] Kajiyama T., Miyamoto A., Kikuchi H., Morimura Y. (1989). Aggregation states and electro-optical properties based on light scattering of polymer/ (liquid crystal) composite films. Chem. Lett..

[33] Liu J.-H., Wu F.-T. (2005). Synthesis of photoisomeric azobenzene monomers and model compound effect on electric-optical properties in PDLC films. J. Appl. Polym. Sci..

[34] Higgins D.A., Hall J.E., Xie A. (2005). Optical microscopy studies of dynamics within individual polymer-dispersed liquid crystal droplets. Acc. Chem. Res..

[35] Lovinger A.J., Amundson K.R., Davis D.D. (1994). Morphological Investigation of UV-Curable Polymer-Dispersed Liquid-Crystal (PDLC) Materials. Chem. Mater..

[36] Jayoti D., Malik P., Singh A. (2017). Analysis of morphological behavior and electro-optical properties of silica nanoparticles doped polymerdispersed liquid crystal composites. J. Mol. Liq..

[37] Kim J., Han J.I. (2014). Effect of cell gap on electro-optical properties of polymer dispersed liquid crystal lens for smart electronic glasses. Electron. Mater. Lett..

[38] Chen C.-C. (2016). Low power consumption and high-contrast light scattering based on polymer-dispersed liquid crystals doped with silver-coated polystyrene microspheres. Opt. Express.

[39] Wu Q., Wang Y. (2017). Low driving voltage ITO doped polymer-dispersed liquid crystal film and reverse voltage pulse driving method. Appl. Opt..

[40] Liu F., Cao H., Mao Q., Song P., Yang H. (2012). Effects of monomer structure on the morphology of polymer networks and the electro-optical properties of polymer-dispersed liquid crystal films. Liq. Cryst..

[41] Schulte M.D., Clarson S.J., Natarajan L.V., Tomlin D.W., Bunning T.J. (2000). The effect of fluorine-substituted acrylate monomers on the electro-optical and morphological properties of polymer dispersed liquid crystals. Liq. Cryst..

[42] Yuan Y., Fan F., Zhao C., Kwok H.-S., Schadt M. (2020). Low-driving-voltage, polarizer-free, scattering-controllable liquid crystal device based on randomly patterned photo-alignment. Opt. Lett..

[43] Yoon W., Choi Y., Lim S., Koo J., Yang S., Jung D., Kang S., Jeong K.-U. (2019). A Single-Step Dual Stabilization of Smart Window by the Formation of Liquid Crystal Physical Gels and the Construction of Liquid Crystal Chambers. Adv. Funct. Mater..

[44] Abdulhalim I., Madhuri P.L., Diab M., Mokari T. (2019). Novel easy to fabricate liquid crystal composite with potential for electrically or thermally controlled transparency windows. Opt. Express.

[45] Koduru H.K., Marino L., Scaramuzza N. (2019). Electro-optics of PDLC films doped with WO3 nanoparticles. AIP Conference Proceedings 2075.

[46] Wu Y., Cao H., Duan M., Li E., Wang H., Yang Z., Wang N., He W. (2017). Effects of a chemically modified multiwall carbon nanotubes on electro-optical properties of PDLC films. Liq. Cryst..

[47] Zhou L., Ma H., Han C., Hu W., Zhang S., Zhang L., Yang H. (2018). A novel light diffuser based on the combined morphology of polymer networks and polymer balls in a polymer dispersed liquid crystals film. RSC Adv..

[48] Oh N.-S., Shin Y.-H., Kang H.-Y., Kwon S.-B. (2017). High performance dye-doped emulsion type PDLC for transmittance variable devices. Mol. Cryst. Liq. Cryst..

[49] Kizhakidathazhath R., Higuchi H., Okumura Y., Kikuchi H. (2018). Effect of polymer backbone flexibility on blue phase liquid crystal stabilization. J. Mol. Liq..

[50] Jeong E.H. (2010). Memory effect of polymer dispersed liquid crystal by hybridization with nanoclay. Express Polym. Lett..

[51] Lee Y.-H., Gou F., Peng F., Wu S.-T. (2016). Hysteresis-free and submillisecond-response polymer network liquid crystal. Opt. Express.

[52] Amundson K., van Blaaderen A., Wiltzius P. (1997). Morphology and electro-optic properties of polymer-dispersed liquid-crystal films. Phys. Rev. E.

[53] Nicoletta F.P., Chidichimo G., Cupelli D., de Filpo G., de Benedittis M., Gabriele B., Salerno G., Fazio A. (2005). Electrochromic Polymer-Dispersed Liquid-Crystal Film: A New Bifunctional Device. Adv. Funct. Mater..

[54] Amudson K. (1996). Electro-optic properties of a polymer-dispersed liquid-crystal film: Temperature dependence and phase behavior. Phys. Rev. E.

[55] Kizhakidathazhath R., Higuchi H., Okumura Y., Kikuchi H. (2017). Weak Anchoring Interface Inducing Acrylate Copolymer Designs for High-Performance Polymer-Stabilized Blue Phase Liquid Crystal Displays. Chemistry.

[56] Rijeesh K., Higuchi H., Okumura Y., Yamamoto J., Kikuchi H. (2017). Liquid crystal anchoring transitions and weak anchoring interface formation at surfaces created by uniquely designed acrylate copolymers. Polymer.

