# Supplementary material for: High-Performance Polymer Dispersed Liquid Crystal Enabled by Uniquely Designed Acrylate Monomer
Source: Polymers (Basel). 2020 Jul 22;12(8):1625. doi: 10.3390/polym12081625 (PMC7466073; doi:10.3390/polym12081625)
Supplement: Supplementary file 1 [file polymers-12-01625-s001.pdf]

High-performance Polymer Dispersed Liquid Crystal Enabled by Uniquely Designed Acrylate monomer

Rijeesh Kizhakidathazhath,*, [1] Hiroya Nishikawa, [1] Yasushi Okumura, [1] Hiroki Higuchi, [1] Hirotsugu Kikuchi *, [1]

[1]Institute for Materials Chemistry and Engineering, Kyushu University, Japan

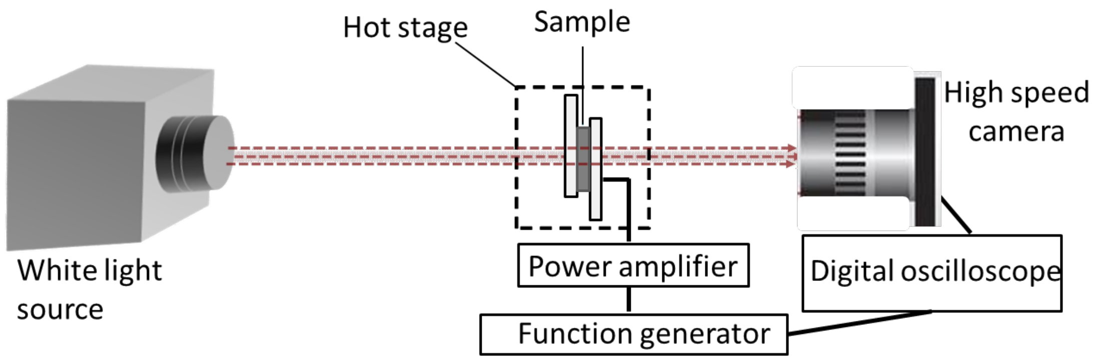

Figure S1: Experimental set up for electro-optical properties measurements of PDLCs.

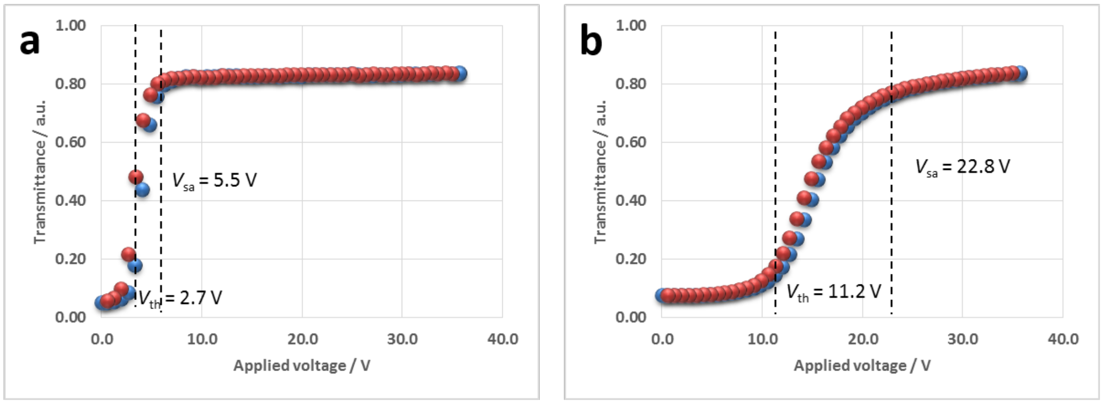

Figure S2: V-T curves for forward (blue circles) and backward (red circles) processes of A3DA-PDLC and reference cell.

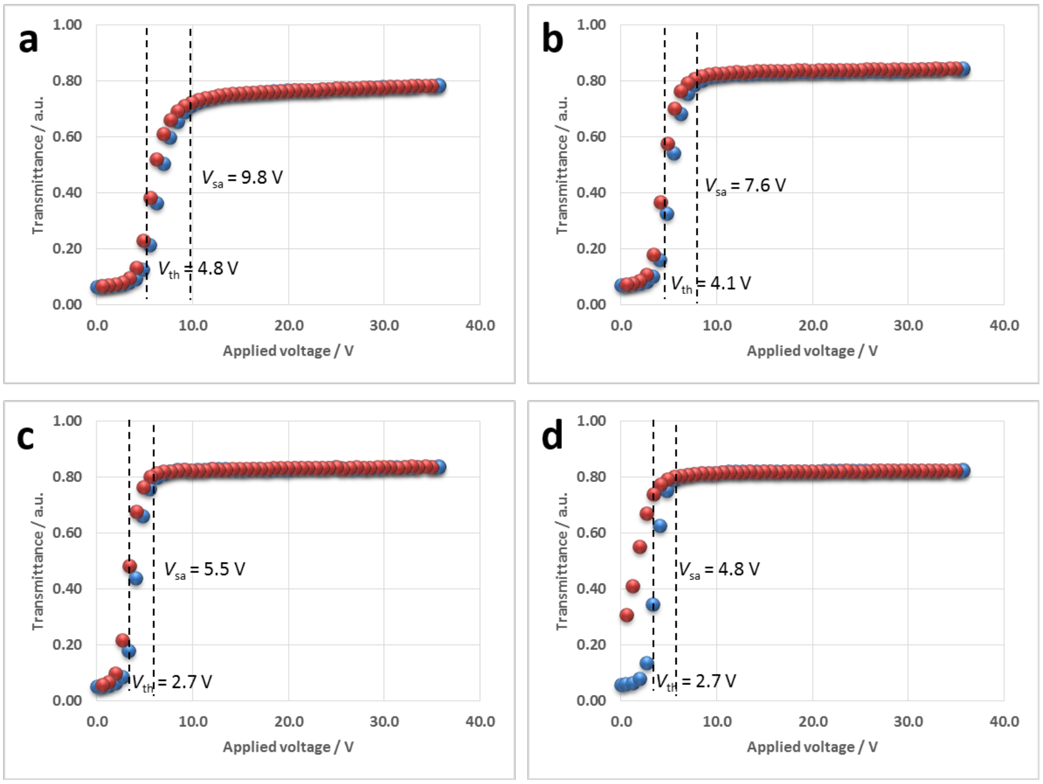

Figure S3: V-T curves for forward (blue circles) and backward (red circles) processes of A3DA-PDLC at various concentrations. a) 8.0 wt. %, b) 8.8 wt. %, c) 9.6 wt. %, d) 11.2 wt. %.

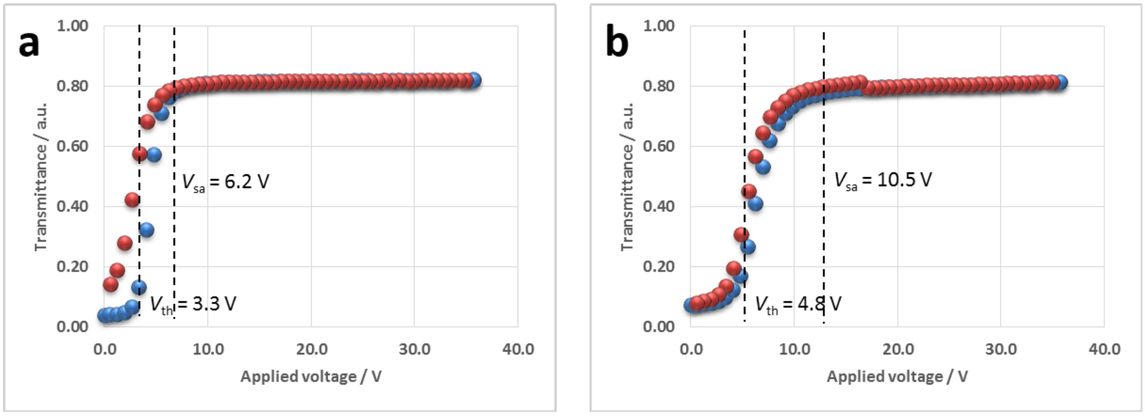

Figure S4: V-T curves for forward (blue circles) and backward (red circles) processes of A0DA-PDLC (a) and DA-PDLC (b).

Table S1: EO properties of PDLCs prepared with A3DA and reference cell at 303 K, λ= 633 nm.

Sample name	R (Std.)	A (11.2 wt. %)	B (9.6 wt. %)	C (8.8 wt. %)	D (8.0 wt. %)
Vth (V)	11.2	2.7	2.7	4.1	4.8
Vmax (V)	22.8	4.8	5.5	7.6	9.8
Contrast ratio	11.30	14.37	16.82	12.22	12.17
Rise time (ms)	0.73	-	7	3.2	2
Fall time (ms)	0.98	-	14	6.7	3

Table S2: EO properties of PDLCs prepared with A3DA, A0DA and DA at 303 K, λ= 633 nm. The monomer mixing ratio was 9.6 wt %.

Monomers	A0DA	A3DA	DA
Vth (V)	3.3	2.7	4.8
Vmax (V)	6.2	5.5	10.5
Contrast ratio	21.10	16.82	11.33
Rise time (ms)	0.95	7	2.6
Fall time (ms)	49	14	9.6

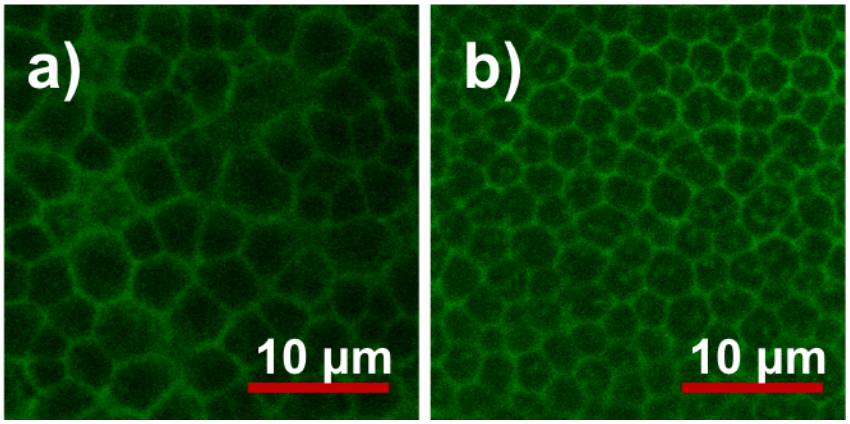

Figure S5: Confocal microscopy images of reference cell (a) and A3DA-PDLC (b) with 0.05 wt% of dye. The images were collected near the film-cover glass interface (UV light exposed area).
